# Supplementary material for: Spin transport of a doped Mott insulator in moiré heterostructures
Source: Nat Commun. 2024 Nov 26;15:10252. doi: 10.1038/s41467-024-54633-z (PMC11599941; doi:10.1038/s41467-024-54633-z)
Supplement: Supplementary file 1 — Supplementary information [file 41467_2024_54633_MOESM1_ESM.pdf]

Supplementary Information for

Spin transport of a doped Mott insulator in moiré heterostructures

Emma C. Regan†, Zheyu Lu†, Danqing Wang†, Yang Zhang, Trithep Devakul, Jacob H. Nie, Zuocheng Zhang, Wenyu Zhao, Kenji Watanabe, Takashi Taniguchi, Sefaattin Tongay, Alex Zettl, Liang Fu*, Feng Wang*

S1. Device information

S2. Doping-dependent spatial-temporal pump-probe technique

S3. Spin transport maps at all hole concentrations

S4. Diffusion-decay model

S5. Doping-dependent spin lifetime

S6. Spin transport maps at high temperatures

S7. Effective *t-J* model in heterobilayer WSe_2/WS_2

S8. Spin diffusion in *t-J* model

S9. Spin diffusion in the high-temperature limit

S10. Spin diffusion in *t-J* model from exact diagonalization

S1. Device information

To fabricate the WSe_2/WS_2 moiré superlattices, we first exfoliate WS_2 and WSe_2 monolayers from bulk crystal onto SiO_2/Si substrates and do polarization-resolved second harmonic generation (SHG) measurements to determine the crystal orientation in each flake [1, 2]. We then assemble the WS_2 and WSe_2 monolayers into a heterostructure with their crystal axes aligned using a polycarbonate (PC) stamp [3]. During the transfer process, the near-zero-degree-twist-angle WSe_2/WS_2 stack is contacted by few-layer graphite (FLG) and sandwiched between two hexagonal boron nitride (hBN) layers with thickness of 15-25 nm. Additional FLG flakes serve as electrostatic gates. The whole heterostructure is released onto a 90 nm SiO_2/Si substrate. Electrodes (100 nm Au with 5 nm Cr adhesion layer) are fabricated using a photolithography system (Durham Magneto Optics, MicroWriter) and an electron-beam deposition system. After fabrication, we again perform polarization-resolved SHG measurements on the monolayer WS_2 and WSe_2 regions within the heterostructure to determine the exact twist angle and on the heterostructure region to distinguish between near-zero and near-sixty-degree samples.

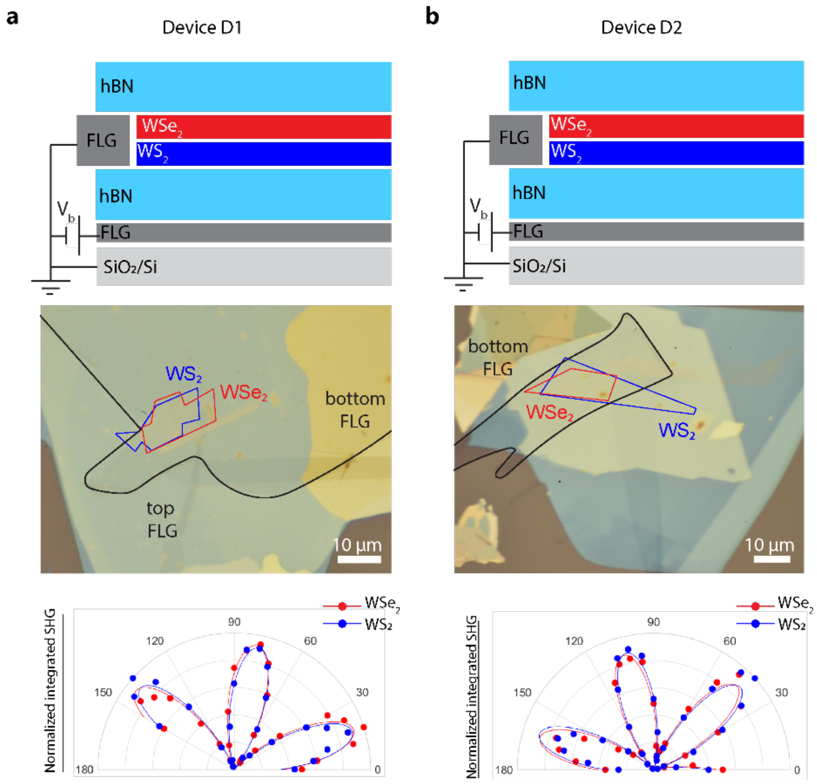

Supplementary Fig. 1 | Device information. Sample schematics (upper panel), optical-microscope images (middle panel), and polarization-resolved SHG data (bottom panel) for the two devices described in this study. In the SHG data, the red (blue) circles correspond to normalized integrated SHG signal at different polarization angles θ for the WSe_2 (WS_2) layers, and the solid lines are the corresponding fits to a $\cos^2(3\theta)$ function. (**a**) Device D1: Near-zero-degree WSe_2/WS_2 heterostructure described in the main text. (**b**) Device D2: Near-zero-degree WSe_2/WS_2 heterostructure described in the main text.

S2. Doping-dependent spatial-temporal pump-probe technique

The spatial-temporal pump-probe technique used in this study (Supplementary Fig. 2) is based on the experiment described in Ref. [4].

A function generator (Siglent SDG6022X) is used to generate two synchronized electronic pulse trains that drive two RF-coupled laser diode modules (Thorlabs LDM56) with center wavelengths of ~690 nm (pump) and ~730 nm (probe). The probe laser wavelength is finetuned to be near resonant with the lowest energy WSe_2 A exciton using the temperature-controlled mount. The pump beam travels through a polarizer and shared quarter waveplate (QWP) to generate a left circularly polarized pump. The probe beam travels through a polarizer, rotating half wave plate (HWP), and the shared QWP to switch between a left- and right-circularly polarized probe. The pump and probe beams are combined with a dichroic mirror and focused onto the sample mounted in a Montana Instruments cryostation using an objective. An additional cylindrical lens in the pump path ensures that the pump beam is focused into a line on the sample. The pump-probe spatial separation is tuned via a piezo-controlled mirror in the pump path. For all pump-probe measurements, the pump power is set such that the photoexcited hole population is small compared to the electrostatically injected hole population.

The reflected beams are spectrally filtered with a 715 nm long-pass filter to isolate the probe, which is then monitored with an avalanche photodiode (APD). The APD output is sent to a Keithley 2400 SourceMeter (measures the static reflectivity, RC) and a SRS864A lock-in amplifier (measures the pump-induced change in the reflectivity, ΔRC). The lock-in amplifier is locked to the frequency of the optical chopper in the pump path.

Doping dependent spin transport measurements are conducted by applying voltages to the graphite gate using Keithley 2400 or 6482 SourceMeters. The charge density, p, is defined using a parallel plate capacitor model:

$$p = \pm \frac{1}{e} \frac{\epsilon_{\mathrm{hBN}} \epsilon_0}{d} (V - V_0)$$

where ϵ_{hBN} is the dielectric constant of hBN (measured as 4.2 ± 0.2 in Ref. [5]), ϵ_0 is the permittivity of free space, d is the thickness of the bottom hBN layer, and V is the voltage applied to the gate. We account for the quantum capacitance (voltage range where charges are not injected) using an offset voltage V_0 for electron and hole doping. The offsets are defined by the voltage where the reflection contrast spectrum begins to change with electron and hole doping.

The moiré density p_0 is defined to correspond to one hole per moiré unit cell and is determined through the relation $p_0 = 1/[L_{\mathrm{M}}^2 \sin(\pi/3)]$ where L_{M} is the moiré superlattice constant. The twist angle (θ) and lattice mismatch ($\delta = (a - a')/a$) between the two layers determines L_{M} via $L_{\mathrm{M}} = a/\sqrt{\delta^2 + \theta^2}$. STM measurements of a near-zero degree WSe_2/WS_2 give $L_{\mathrm{M}} \sim 8$ nm (Ref. [6]), which is consistent with that expected due to the lattice constant mismatch between the layers. Therefore, for zero-degree-twist angle samples, $p_0 = 1.80 \times 10^{12}$ cm^{-2}.

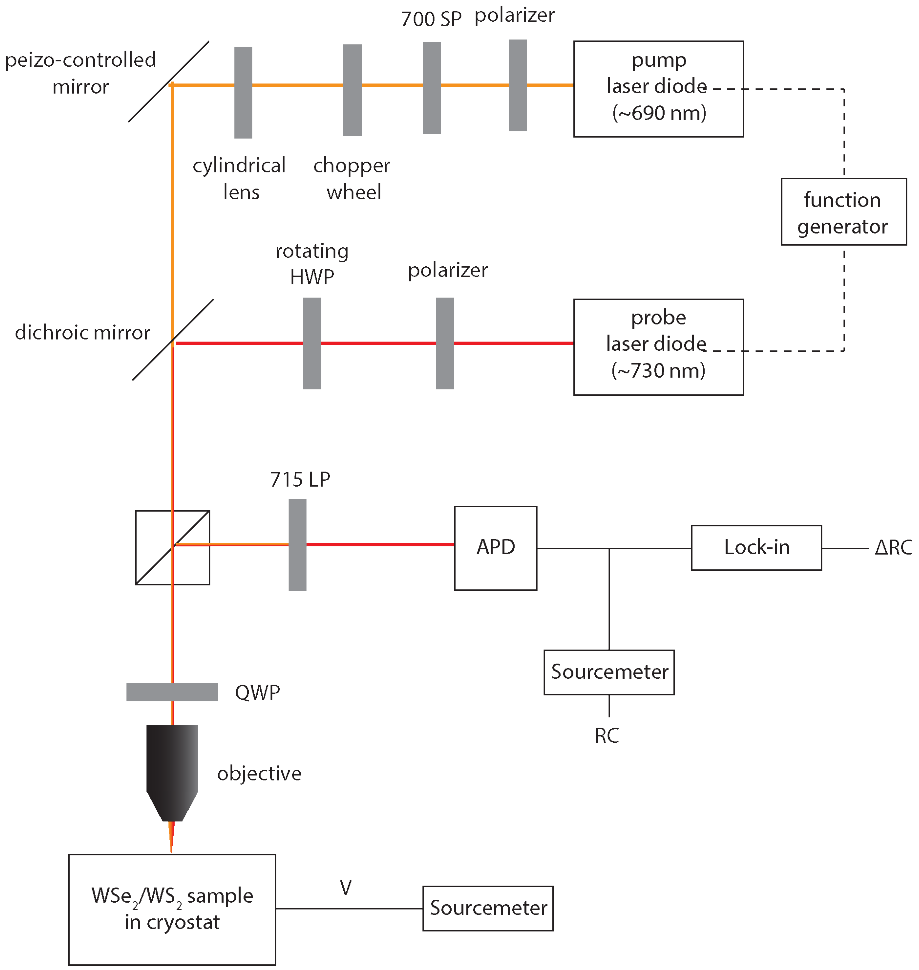

Supplementary Fig. 2 | Experimental setup for spatial-temporal pump-probe measurement.

S3. Spin transport maps at all hole concentrations

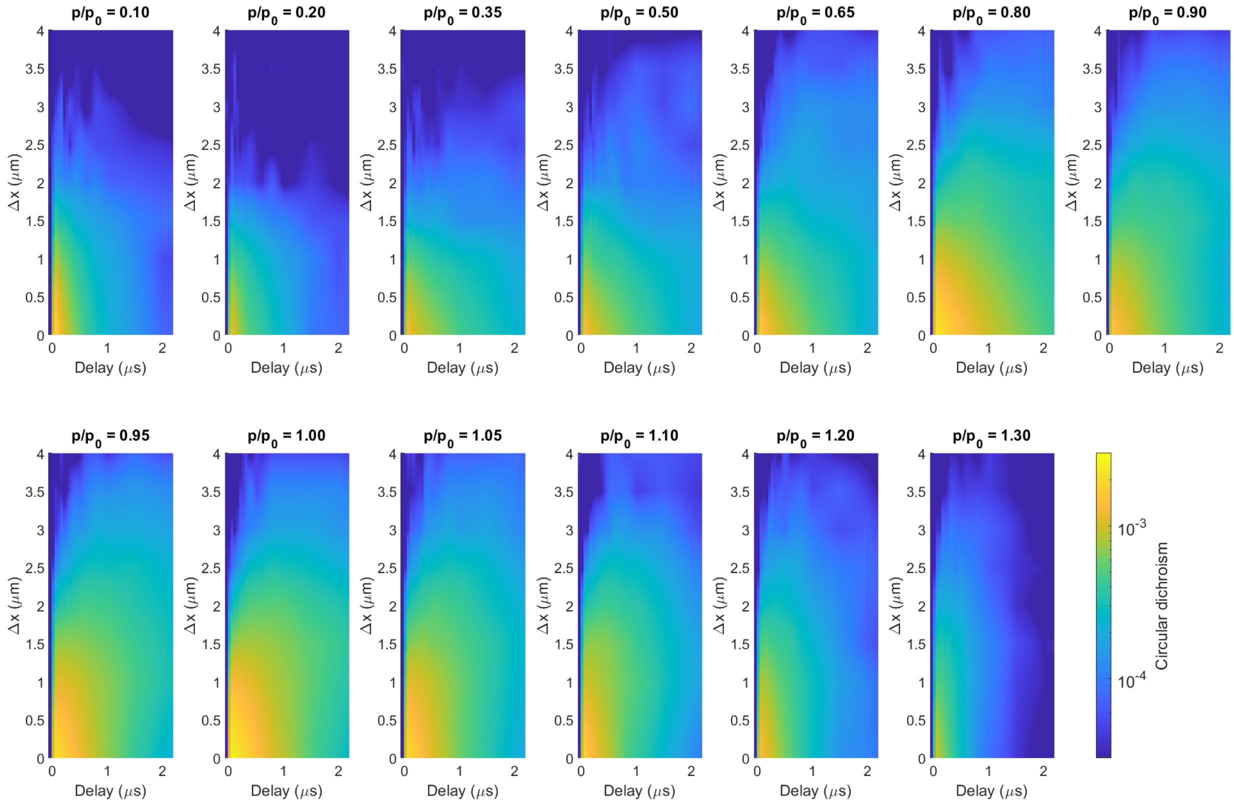

Supplementary Fig. 3 | Spin transport maps in device D1 at all hole concentrations measured. The spatial-temporal evolution of a spin excitation in the WSe_2/WS_2 heterostructure at various hole concentrations, labelled in units of p/p_0. The horizontal and vertical axes represent the temporal and spatial separation between the pump and probe pulses, respectively. The color represents the circular dichroic signal on a log scale. 11 K.

S4. Diffusion-decay model

The pump beam generates a 1D spin excitation, which then diffuses across the sample due to the spatial imbalance in spin density. This behavior can be modeled using a simple diffusion-decay model, as described in detail in Ref. [4]. The density of spin-polarized holes is

$$\Delta p_v(x,t) = \frac{\Delta p_0}{\sqrt{\pi(\sigma_0^2 + 4D_s t)}} e^{-\frac{x^2}{\sigma_0^2 + 4D_s t}} e^{-\frac{t}{\tau}},$$

where Δp_0 is the total number of pump-induced spin-polarized holes, σ_0 is the half width of the pump beam (~1 μm), D_s is the spin diffusion constant, and τ is the spin lifetime. The pump-probe signal is calculated by convolving $\Delta p_v(x, \Delta t)$ with the probe beam intensity.

We fit the spatial-temporal pump-probe data to this model at each hole concentration (Supplementary Fig. 4). In this fitting, we allow for a slowly decaying signal near the noise floor of our experiment (~2×10^{-5}), which accounts for the small background signal at low doping, where both D_s and τ are small. The extracted D_s and τ from this fitting provide quantitative information about the spin diffusion and decay in the superlattice, as described in the main text.

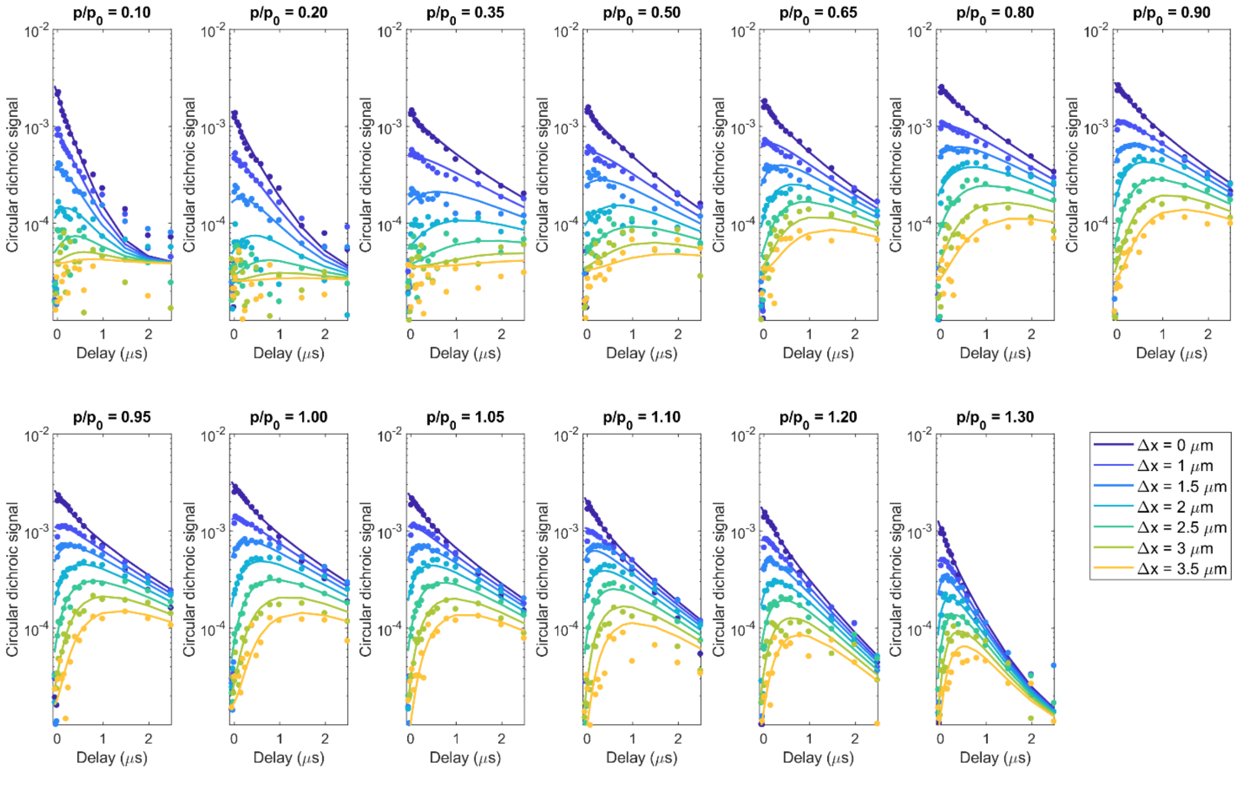

Supplementary Fig. 4 | Fitting spatial-temporal pump-probe data to the diffusion-decay model. Linecuts of the spatial-temporal pump-probe data shown in Supplementary Fig. 3, with the hole concentration labelled in units of p/p_0. For each pump-probe spatial separation (Δx), the dots are data, and the lines are fits to the diffusion-decay model.

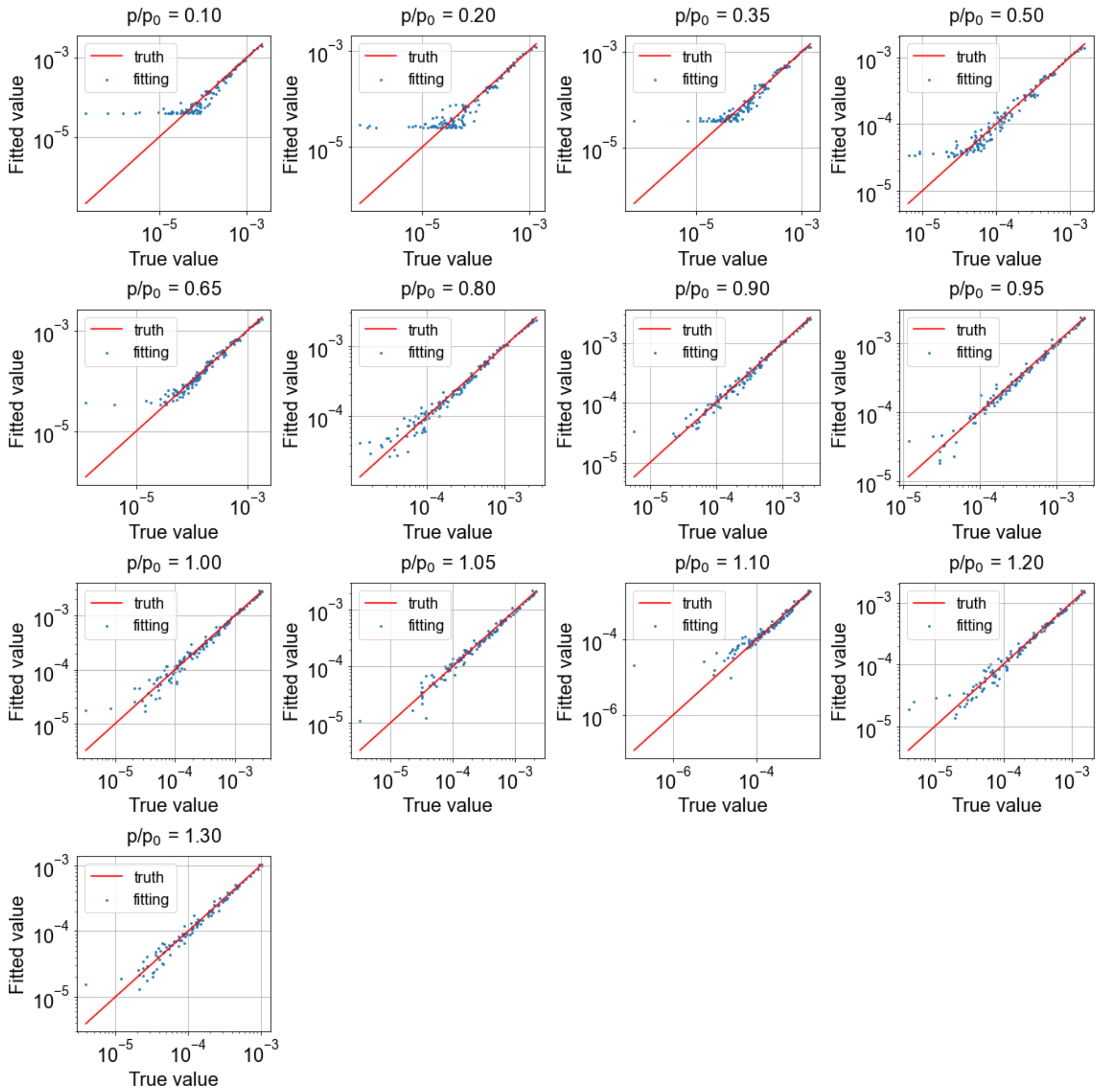

Supplementary Fig. 5 | Visualization of the fitting in Supplementary Fig. 4.

S5. Doping-dependent spin lifetime

Fitting the spatial-temporal pump-probe data from device D1 to a diffusion decay model gives the spin diffusion constant and lifetime at various hole concentrations in the WSe_2/WS_2 moiré superlattice. The spin diffusion constant is discussed in the main text, and the spin lifetime is shown in Supplementary Fig. 6. At low hole concentration, the spin lifetime increases with doping because the spin lifetime becomes decoupled from the charge lifetime [4]. The lifetime is further enhanced at the Mott insulator states, as discussed in Ref. [5], followed by a rapid decrease at higher doping.

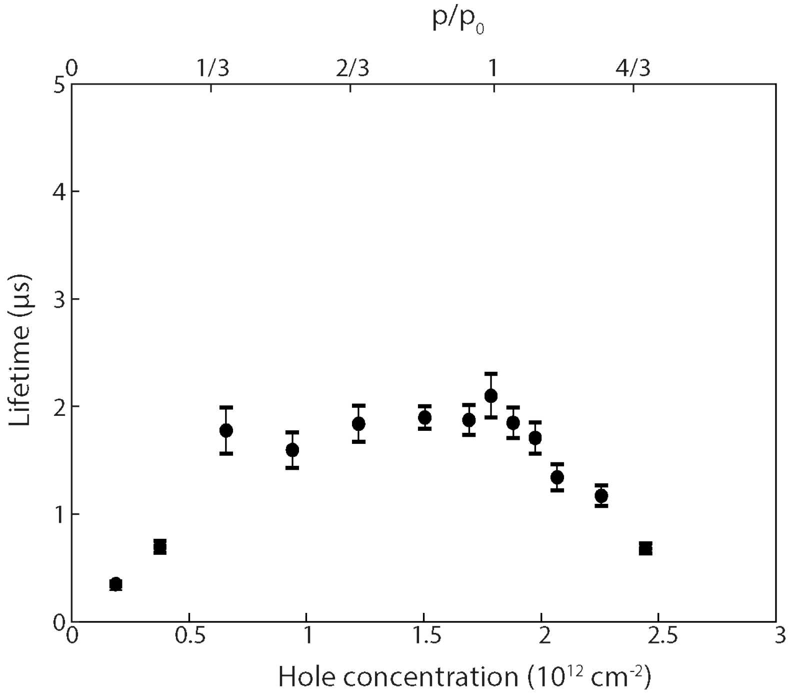

Supplementary Fig. 6 | Doping-dependent spin lifetime in the WSe_2/WS_2 moiré superlattice. Extracted spin lifetime at various initial hole concentrations in device D1 at 11 K.

S6. Spin transport maps at high temperatures

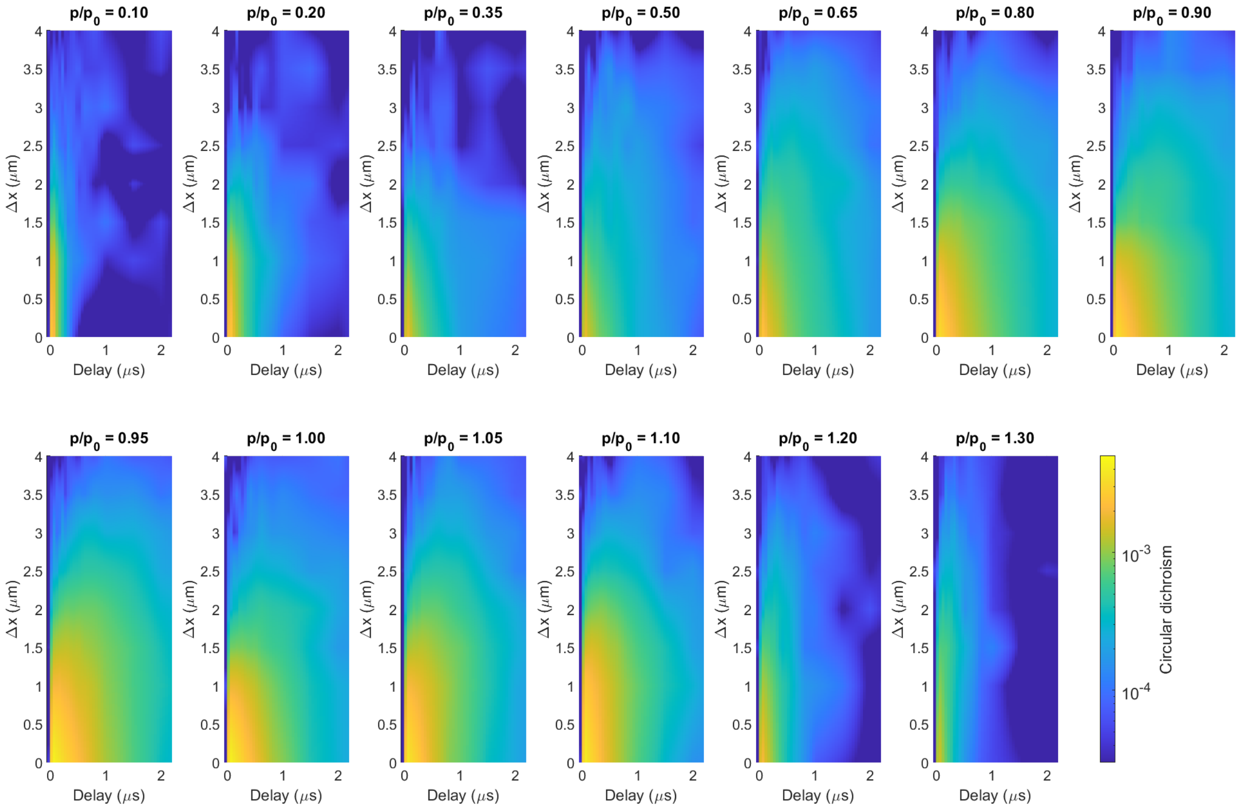

Supplementary Fig. 7 | Spin transport maps in device D1 at 22 K. The spatial-temporal evolution of a spin excitation in the WSe_2/WS_2 heterostructure at various hole concentrations, labelled in units of p/p_0. The horizontal and vertical axes represent the temporal and spatial separation between the pump and probe pulses, respectively. The color represents the circular dichroic signal on a log scale.

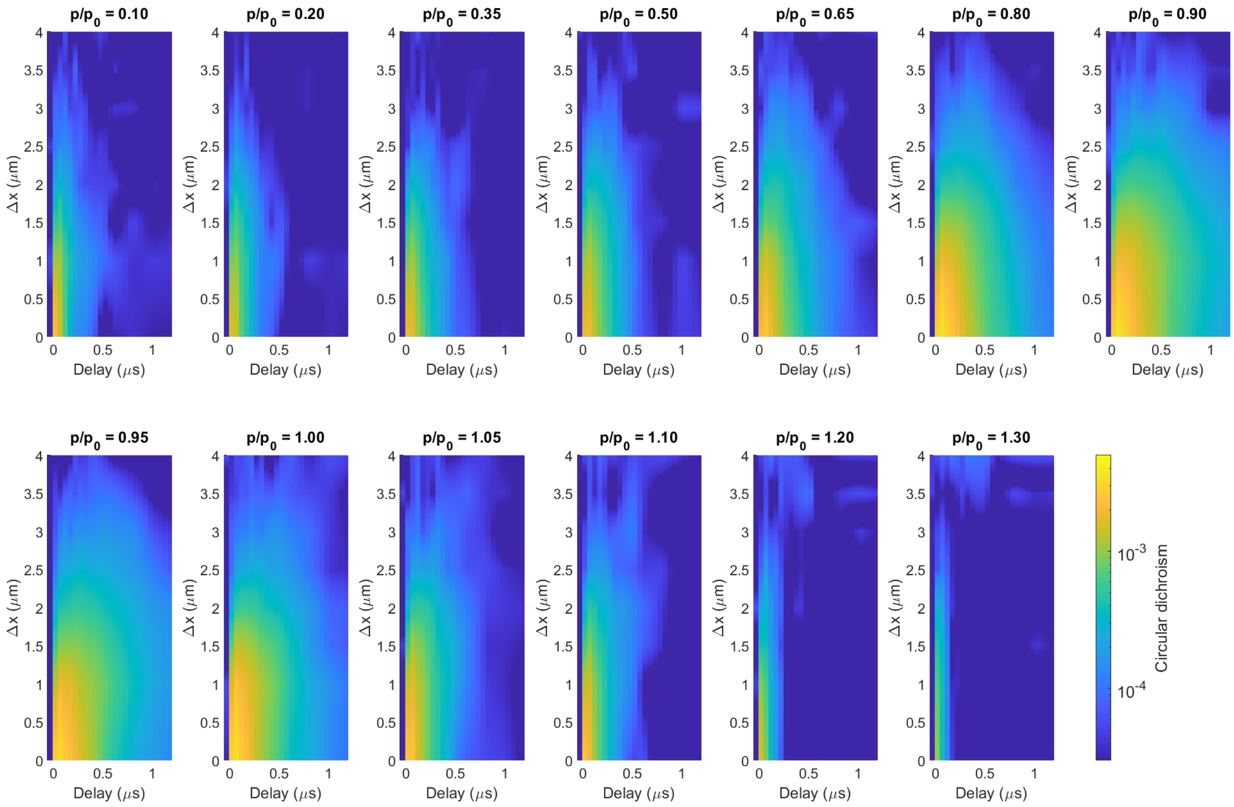

Supplementary Fig. 8 | Spin transport maps in device D1 at 45 K. The spatial-temporal evolution of a spin excitation in the WSe_2/WS_2 heterostructure at various hole concentrations, labelled in units of p/p_0. The horizontal and vertical axes represent the temporal and spatial separation between the pump and probe pulses, respectively. The color represents the circular dichroic signal on a log scale.

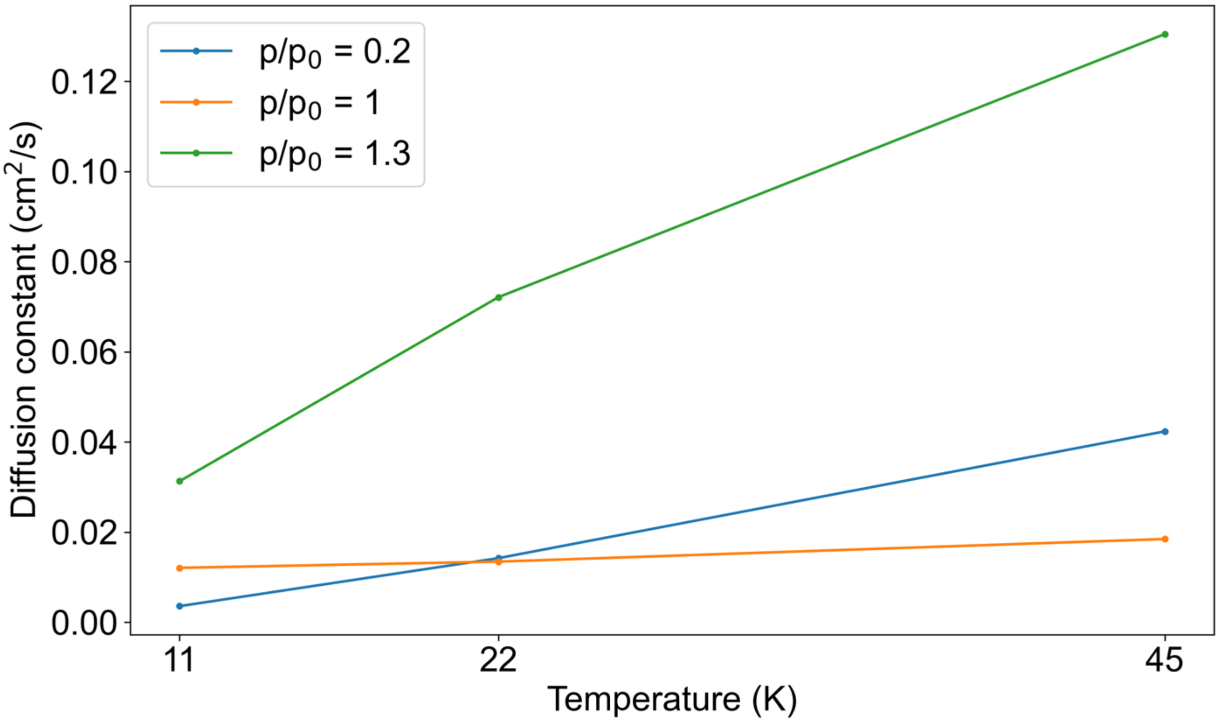

Supplementary Fig. 9 | Comparison of temperature dependence of diffusion constant between different doping.

S7. Effective *t-J* model in heterobilayer WSe_2/WS_2

Hubbard model description and the Mott insulator at $\nu = 1$. The starting point for our analysis of a TMD moiré heterobilayer is the Hubbard model on a triangular lattice [11]:

$$H = -t \sum_{\langle i,j \rangle} \left(c_i^\dagger c_j + h.c. \right) + U \sum_i n_{i\uparrow} n_{i\downarrow}$$

While longer-range interactions are neglected for simplicity, the Hubbard model description is justifiable when the Coulomb interaction is screened by nearby metallic gates. Consistent with this expectation, recent experiments on angle-aligned WSe_2/WS_2 found that with gate screening, the generalized Wigner crystals at fractional fillings $\nu = \frac{1}{3}$ and $\frac{2}{3}$ are quenched, but the Mott insulator at $\nu = 1$ remains robust [12]. For WSe_2/WS_2, a DFT calculation found $t \sim 1$ meV [13], which is much smaller than the on-site Coulomb repulsion U. Note that generalization of H that includes higher moiré bands is necessary to properly describe the charge-transfer insulator (rather than Mott-Hubbard insulator) specific to some TMD heterobilayers including WSe_2/WS_2 [13].

Given the small hoping magnitude, the effective Hubbard model is at the strong coupling region. Without double occupancy, this large-*U* Hubbard model is reduced to the *t-J* model with $J = \frac{4t^2}{U}$.

$$\hat{H} = -t \sum_{\langle ij \rangle, \sigma} \left(c_{i\sigma}^\dagger c_{j\sigma} + \text{ h.c. } \right) + J \sum_{\langle ij \rangle} \left(\vec{S}_i \cdot \vec{S}_j - \frac{n_i n_j}{4} \right)$$

Here $\vec{S}_i$ is the spin-$\frac{1}{2}$ operator given by $\vec{S}_i = \sum_{\sigma\sigma'} c_{i\sigma} \frac{\sigma_{\sigma\sigma'}}{2} c_{i\sigma'}$, where σ is the Pauli matrix.

S8. Spin diffusion in *t-J* model

In this section, we present the general formalism for spin diffusion in *t-J* model. Since the total *z* spin component $S^z = \sum_{\mathbf{r}} S^z(\mathbf{r})$ is conserved in our *t-J* model, we start from the operator-continuity relation [14]:

$$\frac{\partial S^z(\mathbf{r}, t)}{\partial t} + \nabla \cdot \mathbf{j}_s(\mathbf{r}, t) = 0$$

Here $\mathbf{j}_s$ is the spin current operator with spin at *z* direction. At finite temperature, the spin relaxation in the *t-J* model in which the net flow of magnetization from the region of spin injection towards small S^z is a hydrodynamic process, and can be decribed by the diffusion equation:

$$\frac{\partial \langle S^z(\mathbf{r}, t) \rangle}{\partial t} - D_s \nabla^2 \langle S^z(\mathbf{r}, t) \rangle = 0$$

With the linear response, hydrodynamics, and fluctuation-dissipation theorem, we can write the spin-correlation function in momentum space as:

$$
\begin{aligned}
S(\mathbf{k},\omega) &= \langle S^z(\mathbf{k},\omega)S^z(-\mathbf{k},-\omega)\rangle \\
&\approx \frac{2}{1-e^{-\beta\omega}} \times \frac{\omega D_s k^2 \chi}{\omega^2 + (D_s k^2)^2}
\end{aligned}
$$

Here $S(\mathbf{r},t) = \langle S^z(\mathbf{r},t)S^z(0,0)\rangle$ is the spin correlation in the lattice site, β is the inverse temperature, χ is the static spin susceptibility, and D_s is the spin diffusion constant. The brackets refer to the thermodynamic equilibrium averages from the wavefunctions.

We now define the spin conductivity as:

$$
\begin{aligned}
\sigma_x(\mathbf{k},\omega) &= \frac{1-e^{-\beta\omega}}{2\omega} \langle j_x(\mathbf{k},\omega) j_x(-\mathbf{k},\omega)\rangle \\
&= \frac{1-e^{-\beta\omega}}{2\omega} \int_{-\infty}^{\infty} d\,t \sum_{\mathbf{r}} e^{i(\mathbf{k}\cdot\mathbf{r}-\omega t)} \\
&\quad \times \langle j_x(\mathbf{r},t) j_x(0,0)\rangle,
\end{aligned}
$$

where $j_x(\mathbf{r},t)$ is the spin current operator at site $\mathbf{r}$ with spin polarization at z direction and current flow at x direction. With the Heisenberg equation $\frac{d}{dt}S^z_{[r,r']} = i\left[H, S^z_{[r,r']}\right]$, we can write the spin current operator in *t-J* model as:

$$
\begin{aligned}
j_x(\mathbf{r}) = -\frac{i}{2}\sum_{\mathbf{b}} \quad & \left[-t\mathbf{b}_x\left(c^\dagger_{\mathbf{r}\uparrow}c_{\mathbf{r}+\hat{\mathbf{b}}\uparrow} - c^\dagger_{\mathbf{r}\downarrow}c_{\mathbf{r}+\hat{\mathbf{b}}\downarrow}\right)\right. \\
& \left. +J\mathbf{b}_x S^+_{\mathbf{r}} S^-_{\mathbf{r}+\hat{\mathbf{b}}}\right]
\end{aligned}
$$

Here $\mathbf{b}$ sums over six nearest-neighbor bonds in triangular lattice, and $\mathbf{b}_x$ is the projection of bond vector $\mathbf{b}$ at x direction. The spin conductivity can be related to the spin response function $S(\mathbf{k},\omega)$ using operator-continuity relation in $(\mathbf{k},\omega)$ space:

$$
-\omega S^z(\mathbf{k},\omega) + \mathbf{k}\cdot\mathbf{j}(\mathbf{k},\omega) = 0
$$

And we get $\sigma_x(\mathbf{k},\omega) = \frac{1}{2}\omega\left(1-e^{-\beta\omega}\right)\frac{S(\mathbf{k},\omega)}{k^2}$. Combining with spin-correlation function in momentum space, we obtain the Einstein relation between the dc spin conductivity and spin-diffusion constant:

$$
\sigma = \sigma_x(\mathbf{k}=0,\omega=0) = D_\mathrm{s}\chi
$$

And the spin diffusion constant can be calculated via dc spin conductivity and static spin susceptibility $D_\mathrm{s} = \sigma/\chi$.

S9. Spin diffusion in the high-temperature limit

The high temperature limit of the spin diffusion constant in the *t-J* model can be computed via high-temperature expansion and Gaussian approximation, which is valid in the limits $t=0$ or $J=0$ and small hole doping [14]. Following Ref. [14], the spin diffusion constant D_s is expressed as the ratio

$$D_s = \frac{1}{\sqrt{2\pi}}\frac{1}{\chi}\sqrt{\frac{\langle\omega^0\rangle^3}{\langle\omega^2\rangle}}$$

where

$$\langle\omega^0\rangle = \frac{\pi}{T}\sum_{\mathbf{r}}\langle j_x(\mathbf{r})j_x(\mathbf{0})\rangle$$

$$\langle\omega^2\rangle = \frac{\pi}{T}\sum_{\mathbf{r}}\langle [i\,\partial_t j_x(\mathbf{r})][-i\,\partial_t j_x(\mathbf{0})]\rangle$$

where $j_x(\mathbf{r})$ is defined earlier and

$$\partial_t j_x(\mathbf{r})) = i[H, j_x(\mathbf{r})]$$

We compute the high temperature limit of $\langle\omega^0\rangle$ and $\langle\omega^2\rangle$ at a fixed total density, which is accomplished by performing the thermal average at infinite temperature with a fixed fugacity $z = e^{\beta\mu}$. For example, to compute $\langle\omega^0\rangle$ as a function of density, we compute

$$\langle j_x(\mathbf{r})j_x(\mathbf{0})\rangle_z = \mathcal{Z}^{-1}\mathrm{Tr}[j_x(\mathbf{r})j_x(\mathbf{0})z^N]$$

where N is the total particle number operator, $\mathcal{Z} = \mathrm{Tr}[z^N] = (1+2z)^{N_s}$, and N_s is the number of sites in the full system. Sites outside of the support of $j_x(\mathbf{r})j_x(\mathbf{0})$ do not contribute to the infinite temperature expectation value and can be ignored in this calculation. Furthermore, the expectation value is only non-zero when the supports of $j_x(\mathbf{r})$ and $j_x(\mathbf{0})$ have non-zero overlap, hence only a finite number of $\mathbf{r}$ near the origin need to be considered. The average density is related to z by $\nu = 2z/(1+2z) \equiv 1-\delta$, where $0 \le \delta \le 1$ is the hole doping density. We find, for the triangular lattice,

$$\frac{T}{\pi}\langle\omega^0\rangle = \frac{3J^2(\delta^2-2\delta+1)}{16} + \frac{3\delta t^2(1-\delta)}{4}$$

$$\frac{T}{\pi}\langle\omega^2\rangle = \frac{3J^4(-5\delta^3+16\delta^2-19\delta+7)}{32} + \frac{3\delta t^4(\delta^3+4\delta^2-11\delta+6)}{2} + O(t^2J^2, J^3t, \ldots)$$

where the terms of mixed order neglected in $\langle\omega^2\rangle$ do not enter the final expression for D_s in the limits of interest.

Plugging these into our equation for D_s and using the high temperature spin susceptibility $\chi = (1-\delta)/(4T)$, we find

$$\frac{D_s^2}{\pi} = \begin{cases} \dfrac{9\delta^2 t^2}{24-20\delta-4\delta^2} & J = 0 \\ \dfrac{9J^2(1-2\delta+\delta^2)}{16(7-5\delta)} & t = 0 \end{cases}$$

As a check, we verify that in the $\delta = 0$ limit, $D_s = 3\sqrt{7\pi}J/28 \approx 0.502J$ agrees with the high temperature expansion of the triangular lattice Heisenberg model [15].

S10. Spin diffusion in *t-J* model from exact diagonalization

We now use the full exact diagonalization to calculate spin conductivity and static spin susceptibility. Since our model is translational invariant, we divide the full Hamiltonian into different symmetry sectors regarding to particle number, spin quantum number and momentum. Due to the translation symmetry, the $k = 0$ spin conductivity can be written as

$$\sigma(\omega) = \frac{1 - e^{-\beta\omega}}{2N\omega} \int_{-\infty}^{\infty} d\, t e^{-i\omega t} \langle J_x(t) J_x(0) \rangle$$

$$J_x(t) = \sum_{\mathbf{r}} j_x(\mathbf{r}, t)$$

Here N is the number of sites. At dc limit, we obtain the $\sigma(\omega = 0)$ after the integral in the basis of many body wavefunction as

$$\sigma(\omega = 0) = \frac{\pi\beta}{N} \sum_{m,n} e^{-\beta E_n} \langle n|j_x(\mathbf{r}|m\rangle\langle n|j_x(\mathbf{r}|m\rangle\delta(E_n - E_m)$$

where $E_{n,m}$ is the energy for many body wavefunction $\langle n, m|$, $\delta(E_n - E_m)$ is the Dirac delta function, and we choose the broadening parameter according to many-body level spacing. The temperature dependent static spin susceptibility χ is calculated via magnetic field dependent total magnetization from full diagonalization spectrum.

$$\chi(T) = \frac{\partial M(T)}{\partial h}$$

In Supplementary Fig. 7, we show results for the temperature dependent spin diffusion constant for a finite cluster with J = 0 at T = 45K, which is well within the high temperature limit. D_s increases linearly with doping since the spin-current carriers are mobile electrons. From comparison with experimental measurements, we estimate a hopping amplitude of $t = 0.3$meV for $\nu < 1$ and $t = 1.9$meV for $\nu > 1$. The exact diagonalization results agree well with the analytic high temperature expansion.

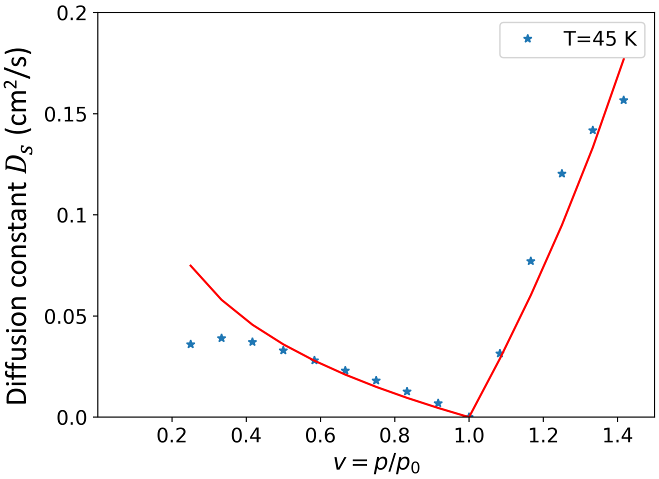

Supplementary Fig. 10 | Filling dependent spin diffusion constant D_s. Density-dependent D_s from $v = 7/12$ to $v = 17/12$ at a 4×3 cluster with translation symmetry. The red line is from high-temperature expansion. The hopping amplitude is fitted from experimental data at T=45 K.

p/p_0	0.10	0.20	0.35	0.50	0.65	0.80	0.90	0.95	1.00	1.05	1.10	1.20	1.30
R^2	0.978	0.972	0.976	0.979	0.985	0.989	0.988	0.980	0.983	0.983	0.970	0.985	0.983
RMSE	3.6e-5	2.5e-5	2.7e-5	2.7e-5	2.8e-5	3.2e-5	3.5e-5	4.3e-5	4.5e-5	3.8e-5	4.5e-5	2.4e-5	1.8e-5

Supplementary Table 1 | Statistical characterization of the fitting in Supplementary Fig. 4.

Supplementary References

1. Kumar, N. *et al.* Second harmonic microscopy of monolayer MoS_2. *Phys. Rev. B* **87**, 161403 (2013).

2. Li, Y. *et al.* Probing symmetry properties of few-layer MoS_2 and h-BN by optical second-harmonic generation. *Nano Lett.* **13**, 3329–3333 (2013).

3. Wang, L. *et al.* One-dimensional electrical contact to a two-dimensional material. *Science* **342**, 614–617 (2013).

4. Jin, C. *et al.* Imaging of pure spin-valley diffusion current in WS_2/WSe_2 heterostructures. *Science* **360**, 893–896 (2018).

5. Regan, E. C. *et al.* Mott and generalized Wigner crystal states in WSe_2/WS_2 moiré superlattices. *Nature* **579**, 359–363 (2020).

6. Li, Hongyuan, *et al.* Imaging moiré flat bands in three-dimensional reconstructed

WSe_2/WS_2 superlattices. *Nature materials* **20**, 945-950 (2021).

7. Wang, Lei, *et al.* Correlated electronic phases in twisted bilayer transition metal dichalcogenides. *Nature materials* **19**, 861-866 (2020).

8. Li, Tingxin, *et al.* Continuous Mott transition in semiconductor moiré superlattices. *Nature* **597**, 350-354 (2021).

9. Tang, Yanhao, *et al.* Simulation of Hubbard model physics in WSe_2/WS_2 moiré superlattices. *Nature* **579**, 353-358 (2020).

10. Xu, Yang, *et al.* Tunable bilayer Hubbard model physics in twisted WSe_2. *arXiv preprint arXiv:2202.02055* (2022).

11. Wu, Fengcheng, *et al.* Hubbard model physics in transition metal dichalcogenide moiré bands. *Phys. Rev. Lett.* **121**, 026402 (2018).

12. Tang, Yanhao, *et al.* Frustrated magnetic interactions in a Wigner-Mott insulator. *arXiv preprint arXiv:2204.08148* (2022).

13. Zhang, Yang, Noah FQ Yuan, and Liang Fu. Moiré quantum chemistry: charge transfer in transition metal dichalcogenide superlattices. *Physical Review B* **102**, 201115 (2020).

14. Bonča, Janez, and Janez Jaklič. Spin diffusion of the t-J model. *Physical Review B* **51**, 16083 (1995).

15. Cowan, B., W. J. Mullin, and E. Nelson. Spin diffusion in 2D and 3D quantum solids. *Journal of Low Temperature Physics* **77**, 181-193 (1989).

16. Davydova, Margarita, Yang Zhang, and Liang Fu. Itinerant spin polaron and metallic ferromagnetism in semiconductor moiré superlattices. *arXiv preprint arXiv:2206.01221* (2022).
